# Large intrinsic anomalous Hall effect in half-metallic ferromagnet Co_3_Sn_2_S_2_ with magnetic Weyl fermions

**DOI:** 10.1038/s41467-018-06088-2

**Published:** 2018-09-11

**Authors:** Qi Wang, Yuanfeng Xu, Rui Lou, Zhonghao Liu, Man Li, Yaobo Huang, Dawei Shen, Hongming Weng, Shancai Wang, Hechang Lei

**Affiliations:** 1 Department of Physics and Beijing Key Laboratory of Opto-electronic Functional Materials & Micro-nano Devices, Renmin University of China, 100872 Beijing, China; 2 Beijing National Laboratory for Condensed Matter Physics and Institute of Physics, Chinese Academy of Sciences, 100190 Beijing, China; 3 School of Physical Sciences, University of Chinese Academy of Sciences, 100190 Beijing, China; 4 State Key Laboratory of Functional Materials for Informatics and Center for Excellence in Superconducting Electronics, SIMIT, Chinese Academy of Sciences, 200050 Shanghai, China; 5 Shanghai Synchrotron Radiation Facility, Shanghai Institute of Applied Physics, Chinese Academy of Sciences, 201204 Shanghai, China; 6 Collaborative Innovation Center of Quantum Matter, Beijing, China

## Abstract

The origin of anomalous Hall effect (AHE) in magnetic materials is one of the most intriguing aspects in condensed matter physics and has been a controversial topic for a long time. Recent studies indicate that the intrinsic AHE is closely related to the Berry curvature of occupied electronic states. In a magnetic Weyl semimetal with broken time-reversal symmetry, there are significant contributions to Berry curvature around Weyl nodes, possibly leading to a large intrinsic AHE. Here, we report the quite large AHE in the half-metallic ferromagnet Co_3_Sn_2_S_2_ single crystal. By systematically mapping out the electronic structure of Co_3_Sn_2_S_2_ both theoretically and experimentally, we demonstrate that the intrinsic AHE from the Weyl fermions near the Fermi energy is dominating. The intrinsic anomalous Hall conductivity depends linearly on the magnetization and can be reproduced by theoretical simulation, in which the Weyl nodes monotonically move with the constrained magnetic moment on Co atom.

## Abstract (web summary)

The large intrinsic anomalous Hall effect (AHE) in magnetic Weyl semimetals is expected but rarely verified experimentally. Here, Wang et al. report large intrinsic AHE with linear dependence on magnetization in a half-metallic ferromagnet Co_3_Sn_2_S_2_ single crystal with Kagome lattice of Co atoms, arising dominantly from the Weyl fermions.

## Introduction

The ordinary Hall effect that arises from the Lorentz force deflecting the moving charge carriers has been well understood^1^. In contrast, the anomalous Hall effect (AHE)^2,3^ has attracted tremendous interests because of the fundamental physics and great potential in technical application^4,5^, but the microscopic origin of AHE is still not fully solved. The key issue is whether the effect is intrinsic or extrinsic. It is now recognized that there are three mechanisms to account for the AHE^6^. One is the extrinsic mechanism related to the scattering affected by the spin–orbit interaction, i.e., the skew scattering and side jump mechanisms^7–9^. The skew scattering model predicts that the $\rho _{{{xy}}}^{{A}}$ is linearly proportional to *ρ*_*xx*_, whereas, the side jump models give $\rho _{{{xy}}}^{\mathrm{A}} \propto \rho _{{{xx}}}^{\mathrm{2}}$. The other is intrinsic Kaplus–Luttinger (KL) mechanism related to spin–orbit interaction of Bloch electronic bands, originally proposed by Karplus and Luttinger, which also gives $\rho _{{{xy}}}^{\mathrm{A}} \propto \rho _{{{xx}}}^{\mathrm{2}}$^10^. Importantly, recent studies indicate that there is an intimate relation between the AHE and the Berry curvature of occupied electronic Bloch states^11–14^.

Recently-discovered topological semimetals (TSMs) are characterized by the topologically robust or symmetry-protected bulk band crossings near the Fermi energy (*E*_F_)^15–18^. Classified by the degeneracy of nodes, Dirac and Weyl semimetals (WSMs) with the 4- and 2-fold degenerate Dirac or Weyl points, respectively, have been theoretically predicted and experimentally confirmed^18–22^. Especially, in a magnetic WSM with broken time-reversal symmetry (TRS), Weyl node can be seen as a magnetic monopole in momentum space^23^, around which there are significant contributions to Berry curvature^24^. Thus, if the position of Weyl nodes are proper, especially close to *E*_F_, and the individual Fermi surface (FS) sheets have nonzero Chern numbers, there should be large intrinsic AHE in magnetic WSMs nearly proportional to the distance between a pair of Weyl nodes with opposite chirality^24^. On the other hand, the half-metallic ferromagnets (HMFMs) can also exhibit AHE, such as ferromagnetic (FM) Heusler alloy Co_2_MnGa, NiMnSb and related intermetallic compounds^25,26^. Different from traditional ferromagnets, the HMFMs have attracted extensive interests because of the (nearly) 100% spin polarization of conduction electrons at *E*_F_^27^. They can be perfectly applied to spintronic devices.

In this work, we study the AHE and electronic structure of HMFM Co_3_Sn_2_S_2_ with kagome layers of Co. As previous experimental results reported on polycrystalline samples, the compound orders ferromagnetically at *T*_C_ = 177 K and the spontaneous magnetic moment is about 0.3 μ_B_/Co^28–30^. By employing systematic first-principles calculations, transport, and angle-resolved photoemission spectroscopy (ARPES) measurements on Co_3_Sn_2_S_2_ single crystals, we reveal the quadratically scaling relationship between anomalous Hall resistivity $\rho _{{{xy}}}^{\mathrm{A}}$ and *ρ*_*xx*_, and the linear behavior between intrinsic anomalous Hall conductivity (AHC) and the magnetization. Furthermore, the agreement between the experimental band structures and theoretical calculations support the existence of magnetic Weyl fermions in Co_3_Sn_2_S_2_. Our findings suggest the main contribution of observed large AHE originates from the intrinsic mechanism, which is intimately related to the Weyl nodes near *E*_F_.

## Results

### Structure and Ferromagnetic state in Co_3_Sn_2_S_2_

**Fig. 1:**
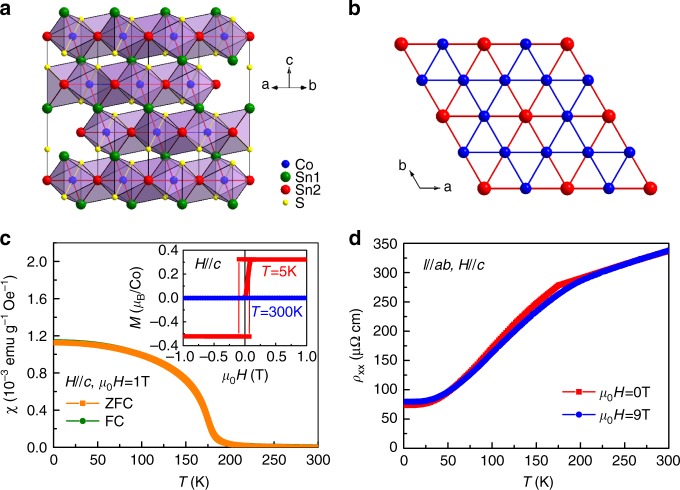
Structure, magnetization and longitudinal resistivity of Co_3_Sn_2_S_2_. **a**, **b** Crystal structure of Co_3_Sn_2_S_2_ and kagome layer made of Co atoms. The small blue and yellow balls represent Co and S atoms, respectively, and the big green and red balls represent Sn atoms at Sn_1_ and Sn_2_ sites, respectively. **c** Temperature dependence of magnetic susceptibility *χ*(*T*) with ZFC and FC modes at *μ*_0_*H* = 1 T for *H*||*c*. Inset: field dependence of magnetization *M*(*μ*_0_*H*) at 5 and 300 K for *H*||*c*. **d** Longitudinal resistivity *ρ*_*xx*_(*T*, *μ*_0_*H*) as a function of temperature *T* at *μ*_0_*H* = 0 and 9 T along the *c* axis

Co_3_Sn_2_S_2_ is crystalized in a hexagonal lattice with space group of $R\bar 3m$ (No. 166). As shown in Fig. 1a, the crystal structure of Co_3_Sn_2_S_2_ is composed of the slabs of CoSn_4_S_2_ octahedra stacking in a hexagonal packing (A-B-C fashion) along the *c* axis. Each Co atom is surrounded by four Sn and two S atoms, forming a distorted octahedron. The Co and S atoms are located at Wyckoff position 9*e* (1/2, 0, 0) and 6*c* (0, 0, 0.219), respectively. The CoSn_4_S_2_ octahedra connect each other along *ab* plane by face-sharing and along *c* axis by corner-sharing. On the other hand, the Co atoms form perfect kagome layer with corner-sharing triangles of Co atoms in Co–Sn layer. There are two kinds of Sn sites. A half of Sn atoms lie in the centers of the kagome hexagons (Sn2 sites, Wyckoff position 3*a* (0, 0, 0)) (Fig. 1b) and another half of Sn atoms located between the Co–Sn bilayers (Sn1 sites, Wyckoff position 3*b* (0, 0, 1/2)), connecting adjacent Co–Sn2 layers. S atoms are located below and above the Co–Sn2 layers. For the plate-like Co_3_Sn_2_S_2_ single crystal, the *c* axis is perpendicular to the crystal surface (Supplementary Fig. 1).

The *χ*(*T*) curves with zero-field-cooling (ZFC) and field-cooling (FC) modes at *μ*_0_*H* = 1 T for *H*||*c* increase rapidly when *T* is below the Curie temperature *T*_C_ (~174 K) (Fig. 1c), well agree with previous results in the literatures^28–30^. Moreover, the ZFC and FC *χ*(*T*) curves overlap each other very well, suggesting that magnetic domains have aligned with the direction of external field when *μ*_0_*H* = 1 T. The field dependence of magnetization *M*(*μ*_0_*H*) at *T* = 5 K for *H*||*c* further confirms the ferromagnetism at low temperature (inset of Fig. 1c). The *M*(*μ*_0_*H*) curve shows a pronounced hysteresis. The hysteresis loop is square and changes its direction at very little coercive field *μ*_0_*H*_*c*_ close to 0.08–0.1 T. The saturated magnetization *M*_s_ is about 0.3 μ_B_/Co, consistent with previous results^28,30^. In contrast, when $T \gg T_{\mathrm{C}}$, the hysteresis behavior vanishes and the *M*(*μ*_0_*H*) curve only shows a paramagnetic behavior (inset of Fig. 1c).

As shown in Fig. 1d, the zero-field longitudinal resistivity *ρ*_*xx*_(*T*, 0) of Co_3_Sn_2_S_2_ shows a metallic behavior in the whole measuring temperature range. A kink can be clearly observed in the *ρ*_*xx*_(*T*, 0) curve around *T*_C_. The resistivity decreases rapidly with decreasing temperature below *T*_C_ due to the decrease of spin disorder scattering. When the external magnetic field is applied (*μ*_0_*H* = 9 T), the metallic behavior of *ρ*_*xx*_(*T*) has almost no change, but the kink of *ρ*_*xx*_(*T*) near *T*_C_ becomes smooth and the staring temperature where the *ρ*_*xx*_(*T*) curve changes the slope shifts to higher temperature.

### Calculated electronic structure, Weyl nodes and AHC

**Fig. 2:**
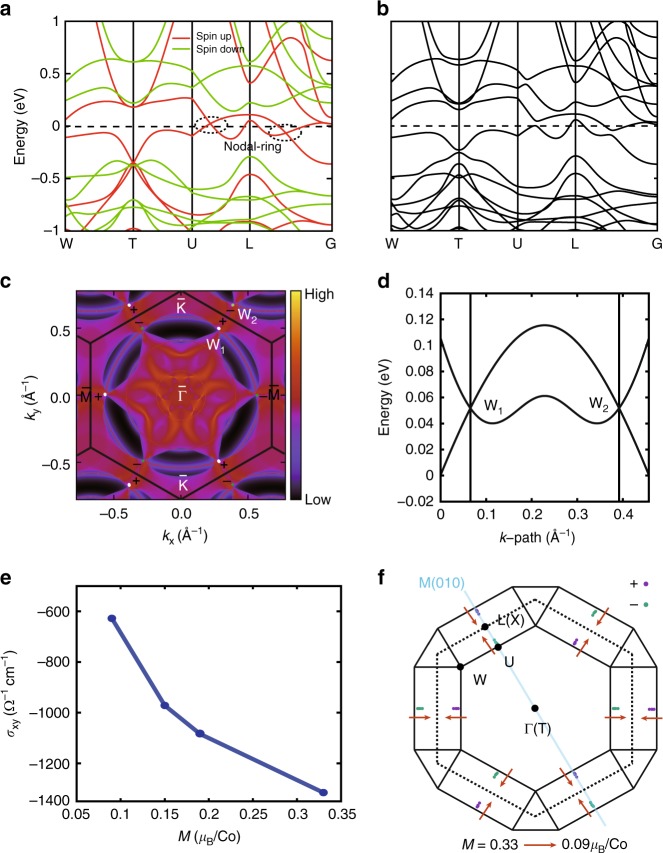
Band structure obtained from first-principle calculations. **a** The spin-resolved band structure without SOC. **b** The band structure with SOC included. The definition of high-symmetry points are shown in Fig. 3a. **c** Fermi surface on the (001) surface cutting on the energy of WPs. The white and green dots represent the projected WPs with positive and negative chirality respectively. **d** The energy dispersion along the direction connecting the WPs W1 and W2 in **c**. **e** The intrinsic anomalous Hall conductivity with the magnetic momentum of 0.09, 0.15, 0.19, and 0.33 μ_B_/Co at 0 K. **f** The evolution of WPs in the BZ with the magnetic momentum decreased from 0.33 to 0.09 μ_B_/Co. The directions of the evolution are indicated by the red arrows. The *E*_F_ is set to 0

The results of the first principle calculation indicate the half-metallic feature of Co_3_Sn_2_S_2_ at FM state (Fig. 2a) and reveal that the bands dispersion near the *E*_F_ are contributed mainly by the 3*d* orbitals of Co with the polarized magnetic momentum about 0.33 μ_B_/Co irrespective of the presence of spin–orbit coupling (SOC). Co_3_Sn_2_S_2_ belongs to the type *I*_*A*_ half metallic ferromagnets and the spins of all electrons are fully polarized along the up direction (defined in *c* axis)^31^. When the SOC is absent, the valence and conductance bands inverse near the *L* point and form a nodal ring (signed by the dashed circles in Fig. 2a), which is protected by the mirror symmetry of the plane M(010) shown in Fig. 2f. When the SOC is further included, it will decay into a pair of Weyl points (WPs) with opposite chirality off the high-symmetry lines (Fig. 2b). The precise positions of the WPs have been obtained by calculating the Wilson-loop evolution^32^ on a much denser *k*-grid in the Brillouin zone (BZ). The evolution of the Wannier charge center (WCC) on a sphere that enclosing a WP in Brillion Zone is demonstrated in Supplementary Fig. 2. There are three pairs of WPs in the BZ in total related by *C*_3_ rotation symmetry and inversion symmetry, as illustrated in Fig. 2c and Supplementary Table 1. From the energy dispersion along the direction connecting the WPs of W1 and W2, the splitting distance of them is about 0.3 Å^−1^ (Fig. 2d), which is far enough to guarantee the robustness of the WPs. We have also calculated the Fermi surface (FS) of (001) surface at the energy of WPs with Green function method^33,34^. As shown in Fig. 2c, the pairs of the WPs with opposite chirality projected on (001) surface are indicated by green and white dots, respectively. Since all of the WPs are mixed with bulk states, it is not easy to clarify the details of how the Fermi arcs connecting the WPs although there are arc-like FSs with large weight of local density of states on surface and preserving *C*_3_ rotation symmetry. In order to distinguish the topological surface states in Fig. 2c, we have redefined a *k*-dependent Fermi energy *E*_F_(*k*) = *α*[*E*_*N*_(*k*) + *E*_*N* + 1_(*k*)]/2, where 0 ≤ *α* ≤ 1. *E*_*N*_(*k*) is the low branch of the two bands crossing at the Weyl nodes and *E*_*N* + 1_(*k*) is the up branch. Thus, if *α* = 1, the *k*-dependent *E*_F_(*k*) will lead to an ideal Weyl semimetal state with and only with Weyl nodes appearing at the Fermi level. When *α* ranges from 0.1 to 0.9 (as shown in Supplementary Fig. 3), we can gradually remove the projected bulk state and identify the Fermi-arc patterns from the other topologically trivial surface states. In Supplementary Fig. 4, the calculations using unrelaxed experimental crystal structure are also shown. The Weyl nodes and magnetic properties are the same as those calculated from relaxed structure. But the WPs and Fermi arcs altered slightly and separated from the projected bulk states. Both the Fermi arcs and the corresponding topological surface states can be identified.

The existence of WPs implies that there would be a large intrinsic AHE in Co_3_Sn_2_S_2_. The AHC *σ*_*xy*_ obtained from the integral of Berry curvature along *k*_*z*_ in the BZ has a very large value of about −1310 Ω cm^−1^ at 0 K (Fig. 2e). Moreover, because the time-reversal symmetry is broken in the FM state, the AHE has a direct relation with the intrinsic magnetic momentum. We constrained the local magnetic momentum as 0.09, 0.15, 0.19, and 0.33 μ_B_/Co and as illustrated in Fig. 2e, the AHC decreases almost linearly with the decreasing of magnetic momentum. Meanwhile, the WPs evolve monotonically with the magnetic momentum decreasing from 0.33 to 0.09 μ_B_/Co as indicated by the red arrows in Fig. 2f. The WPs with opposite chirality approach each other along *k*_*z*_ direction when the spin polarization is reduced. In addition, the AHC of magnetic Weyl semimetals can also be related to the distance of WPs. We can estimate the intrinsic AHC by the formula of *σ*_*xy*_ = *Ke*^2^/4*π*^2^^24^, where *K* is the distance of WPs with opposite chirality along the direction of magnetization. The result also confirms the decrease of intrinsic AHC with decreasing magnetization strength (Supplementary Fig. 6). However, before the pair of Weyl nodes meet each other to annihilate, the two bands composing them become spin degenerate with their time-reversal symmetric partners due to Kramer degeneracy in paramagnetic state.

### Experimental electronic structure observed by ARPES measurements

**Fig. 3:**
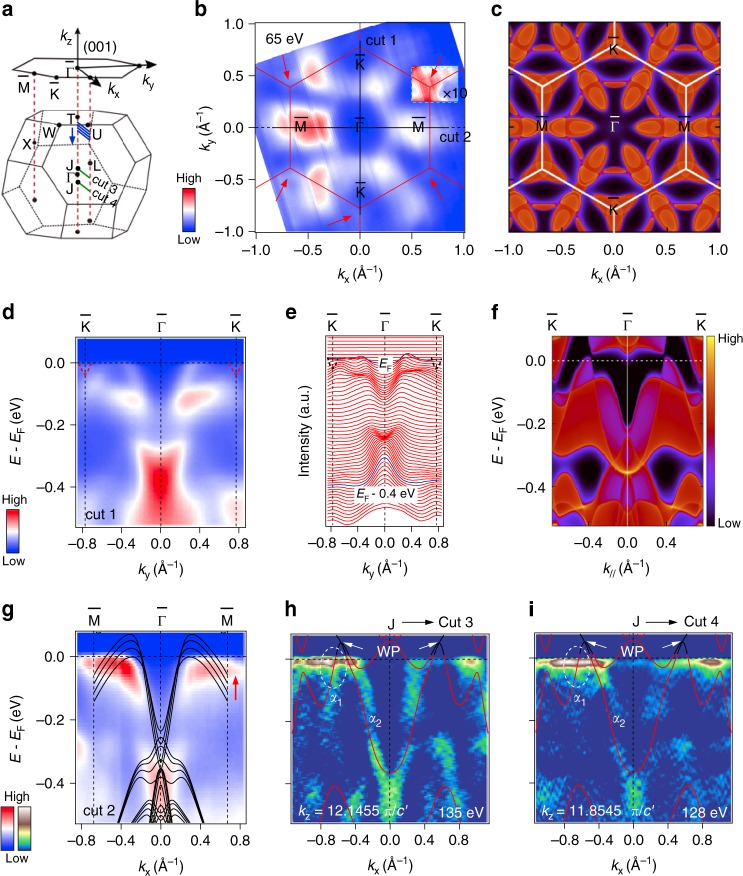
Near-*E*_F_ ARPES spectra compared with first-principle calculations of Co_3_Sn_2_S_2_. **a** Schematic primitive BZ and 2D projected BZ of the (001) surface. Blue lines parallel to the $\overline {\mathrm{\Gamma }} - \overline M$ direction indicate the momentum locations of calculated band structures in **g**, with the *k*_*z*_ momenta of 0.96, 0.92, 0.88, and 0.84 *π* from top to bottom, respectively. Cuts 3 and 4 illustrate the momentum locations of the predicted WPs, which are parallel to the $\overline {\mathrm{\Gamma }} - \overline M$ direction in the *k*_z_ = ± 0.1455 *π* planes. **b** Constant energy ARPES image obtained by integrating the spectral weight within *E*_F_ ± 10 meV recorded with 65 eV photons. Cuts 1 and 2 indicate the momentum locations of the experimental band structures in **d**–**i**. Red lines represent the (001) surface BZs. Red arrows indicate the triangle-shaped FSs centered at $\overline K$ points in **c**. The intensity within the cyan dashed rectangle has been multiplied by 10. **c** Projections of calculated bulk FSs on the (001) surface. White lines represent the (001) surface BZs. **d**, **e** ARPES intensity plot and corresponding momentum distribution curves along the $\overline K - \overline {\mathrm{\Gamma }} - \overline K$ direction [cut 1 in **b**], respectively. Red and black dashed curves illuminate the electron bands forming the triangle-shaped FSs centered at $\overline K$ points. **f** Projections of calculated bulk bands along the $\overline K - \overline {\mathrm{\Gamma }} - \overline K$ direction. **g** ARPES intensity plot along the $\overline M - \overline {\mathrm{\Gamma }} - \overline M$ direction [cut 2 in **b**]. Black curves represent the calculated bulk bands along the four directions indicated by blue lines in **a**, where the bands from bottom to top correspond to the cuts from top to bottom, respectively. **h**, **i** Second derivative intensity plots along cuts 3 and 4 recorded at *hν* = 135 and 128 eV (*k*_z_ = ± 0.1455 *π*), respectively, with corresponding bulk calculations (superimposed red curves). The band crossings for WPs are highlighted by black curves. The crossing-*E*_F_ features of the *α*_1_ bands are marked out by white dashed ellipses

In order to further uncover the electronic origin of AHE in Co_3_Sn_2_S_2_, we performed ARPES experiments on the (001) surface of the samples. The overall band structures are summarized in Fig. 3. The experimental FSs in Fig. 3b are in good agreement with the projections of calculated bulk FSs on the (001) surface in Fig. 3c. The spindle-shaped FSs centered at $\overline M$ points, the triangle-shaped FSs centered at $\overline K$ point, and the ring-like ones connecting the spindle-shaped FSs are all captured by ARPES. Specifically, the agreement of the spindle-shaped FSs is significant since the projection of bands on the (001) surface that creates the WPs are located along the $\overline {\mathrm{\Gamma }} - \overline M$ direction, i.e., the (001)-surface projections of WPs W1 and W2 are located along this direction as illustrated in Fig. 2c, although the WPs are buried into bulk continuum states at the chemical potential.

The detailed near-*E*_F_ band structures along the $\overline {\mathrm{\Gamma }} - \overline K$ and $\overline {\mathrm{\Gamma }} - \overline M$ directions are presented in Fig. 3d–g. We first focus on the results along the $\overline K - \overline {\mathrm{\Gamma }} - \overline K$ direction (Fig. 3d–f), the projections of calculated bulk bands along this direction in Fig. 3f well reproduce the experimental band features in Fig. 3d, e, i.e., the hole bands at near half of $\overline {{\mathrm{\Gamma }}K}$ corresponding to the ring-like FSs and the electron bands (dashed curves in Fig. 3d, e) at $\overline K$ points for the triangle-like FSs, whose intensities are relatively weak due to the matrix element effect. The band structures along the $\overline M - \overline {\mathrm{\Gamma }} - \overline M$ direction are displayed in Fig. 3g. One can obtain that the observed band dispersions are consistent with calculated bulk bands close to the *k*_z_ ~ *π* plane, as illustrated by black curves in Fig. 3g. Indicated by the four blue cuts in Fig. 3a, these bulk bands are located in the *k*_*x*_−*k*_*z*_ plane with *k*_*y*_ = 0 and *k*_*z*_ = 0.96, 0.92, 0.88, and 0.84 *π* from bottom to top, respectively, which are parallel to the $\overline {\mathrm{\Gamma }} - \overline M$ direction. This consistency demonstrates the electronic states at high-symmetry *k*_*z*_ planes (*k*_z_ = *π* here) have main contributions to the ARPES spectra in the frame of *k*_*z*_ broadening effect^35–37^.

Based on the first-principles calculations, the WPs are not located on the high-symmetry *k*_*z*_ planes, they are embedded in the *k*_*z*_ = ±0.1455 *π* planes instead. By performing detailed photon-energy-dependent measurements in Supplementary Fig. 7, we can determine the specific *k*_*z*_ positions of predicted WPs. As shown in Supplementary Fig. 7a, the band dispersions along the $\overline {\mathrm{\Gamma }} - \overline K$ direction show periodic modulation when varying the photon energy. The inner potential (*V*_0_) is thus estimated to be 10 eV. We illustrate the *k*_*z*_ = ±0.1455 *π* planes, where the Weyl fermions with opposite chirality are located, respectively, as blue curves in Supplementary Fig. 7b. To demonstrate the existence of WPs without any interference from possible trivial surface states, we utilize the photons with higher energies to record the band structure. The energies of 138 and 125 eV correspond to the *k*_*z*_ = ±0.1455 *π* planes in the neighbor BZ to that in Supplementary Fig. 7b, respectively. The measured second derivative intensity plots along cuts 3 and 4 (Fig. 3a), which are parallel to the $\overline {\mathrm{\Gamma }} - \overline M$ direction in the *k*_z_ = ±0.1455 *π* planes, are displayed in Fig. 3h, i, respectively. The overlaid bulk band calculations well reproduce the experimental results, especially the *α*_1_ and *α*_2_ bands forming the WPs. The intensity asymmetry of the *α*_1_ band in opposite *k*_*x*_ regions derives from the matrix element effect. Since the cone nature of the Weyl dispersion is an important character proving the realization of massless Weyl fermions, we perform a FS mapping with the photon energy of 135 eV, and study the evolution of band structures gradually off the $\overline {\mathrm{\Gamma }} - \overline M$ direction. As shown in Supplementary Fig. 8b, c, when measuring off the *k*_*y*_ = 0 line, the slopes of the near-*E*_F_ band dispersions (*α*_1_ and *α*_2_) forming the Weyl nodes decrease monotonically. This evolution is in accord with a cone nature within energy–momentum space. We would like to stress that, although the predicted WPs are located at ~50 meV above *E*_F_, the good agreement between theory and experiment, including the two specific *k*_z_ positions of WPs and the band structures forming the WPs below *E*_F_, combined with the valid Weyl–cone structure in experiment, could plausibly demonstrate the existence of Weyl fermions in Co_3_Sn_2_S_2_.

### AHE of Co_3_Sn_2_S_2_

**Fig. 4:**
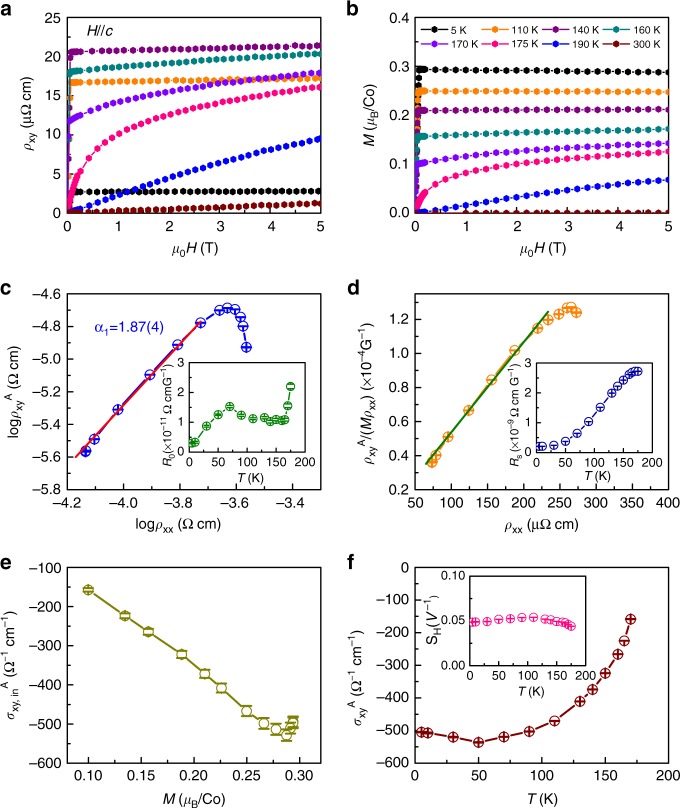
AHE of Co_3_Sn_2_S_2_. **a**, **b** Hall resistivity *ρ*_*xy*_(*μ*_0_*H*) and *M*(*μ*_0_*H*) as a function of magnetic field at various temperatures for *H*||*c*, respectively. **c** Plot of log $\rho _{{{xy}}}^{{A}}(T)$ vs. log *ρ*_*xx*_(*T*). The red solid line is the fit using the relation $\rho _{{{xy}}}^{\mathrm{A}} \propto \rho _{{{xx}}}(T)^\alpha$. **d** Plot of $\rho _{{{xy}}}^{\mathrm{A}}{\mathrm{/}}\left( {M\rho _{{{xx}}}} \right)$ vs. *ρ*_*xx*_. The green solid line is the linear fit when *ρ*_*xx*_ ≤ 250 μΩ cm. Insets of **c** and **d** show the temperature dependence of *R*_0_(*T*) and *R*_s_(*T*) at *T* ≤ *T*_C_, respectively. **e** Intrinsic AHC $\sigma _{{{xy,\mathrm{in}}}}^{\mathrm{A}}(T)$ as a function of *M*. **f** AHC $\sigma _{{{xy}}}^{\mathrm{A}}(T)$ as a function of temperature. Inset: temperature dependence of scale factor *S*_H_(*T*). Error bars in **c**–**f** are the standard deviations of the fits when determining the $\rho _{{{xy}}}^{\mathrm{A}}$ (Supplementary Note 1)

Next, we move to the AHE of Co_3_Sn_2_S_2_. As shown in Fig. 4a, the Hall resistivity *ρ*_*xy*_ of Co_3_Sn_2_S_2_ at high temperature shows the linear field dependence. When *T* is close to *T*_C_, the *ρ*_*xy*_(*μ*_0_*H*) curve starts to bend significantly at low field region. For $T \ll T_{\mathrm{C}}$, the *ρ*_*xy*_(*μ*_0_*H*) curve increases steeply at low field and then becomes weakly field-dependent at high field (above ~0.1 T). The field dependence of *ρ*_*xy*_(*μ*_0_*H*) resembles those of *M*(*μ*_0_*H*) (Fig. 4b), typical ones for ferromagnets. But the saturation values follow opposed temperature dependencies at $T \ll T_{\mathrm{C}}$. When the saturation magnetization increases with decreasing temperature, the saturation value of the *ρ*_*xy*_(*μ*_0_*H*) decreases below *T*_C_. The maximum saturated value of *ρ*_*xy*_(*μ*_0_*H*) is ~21 μΩ cm at *T* = 140 K. We also measured the *ρ*_*xy*_(*μ*_0_*H*) of several crystals at the same temperature. They exhibit similar values, spanning between 19.59 and 24.22 μΩ cm at *μ*_0_*H* = 5 T (Supplementary Fig. 9). The standard deviation of determined sample thickness is also below 5% (Supplementary Fig. 10). Moreover, the *ρ*_*xy*_(*μ*_0_*H*) at various field directions are also measured. When the magnetic field is titled away from the *c* axis of Co_3_Sn_2_S_2_ crystal up to ±30°, the change of *ρ*_*xy*_(*μ*_0_*H*) is small and the [*ρ*_*xy*_(*μ*_0_*H*)(30°) − *ρ*_*xy*_(0°)]/*ρ*_*xy*_(0°) at 5 T is only about 1% (Supplementary Fig. 11). All of these results clearly indicate that the experimental *ρ*_*xy*_(*μ*_0_*H*) is reproducible and reliable.

It is known that the Hall resistivity *ρ*_*xy*_ in ferromagnets arises from two parts^4^, *ρ*_*xy*_ = $\rho _{{{xy}}}^{\mathrm{O}} + \rho _{{{xy}}}^{\mathrm{A}}$ = *R*_0_*B* + 4*πR*_s_*M*, where $\rho _{{{xy}}}^{\mathrm{O}}$ is the normal Hall resistivity due to the Lorentz force, $\rho _{{{xy}}}^{\mathrm{A}}$ is the anomalous Hall resistivity, *R*_0_ is the ordinary Hall coefficient, and *R*_s_ is the anomalous Hall coefficient. From the *ρ*_*xy*_(*μ*_0_*H*) and *M*(*μ*_0_*H*) curves (Supplementary Fig. 12 and Supplementary Note 1), the *R*_0_ and *R*_s_ can be determined. The *R*_0_ is positive at entire  temperature region (inset of Fig. 4c), indicating that the dominant carrier is hole-type. The apparent charge carrier density *n*_a_ can be deduced using the relation of *n*_a_ ~ −1/|*e*|*R*_0_ (Supplementary Fig. 13), and it reaches 2.1 × 10^22^ cm^−3^ at 5 K, corresponding to about 0.7 carriers per formula unit of Co_3_Sn_2_S_2_. The *R*_s_(*T*) is also positive but the absolute value is much larger than *R*_0_ (inset of Fig. 4d). It increases monotonically with increasing temperature. The obtained *R*_s_ value is 2.7 × 10^−9^ Ω cm G^−1^ at 170 K, which exhibits an enhancement of two orders of magnitude when compared with the conventional itinerant ferromagnets, such as pure Fe and Ni^38,39^.

## Discussion

The relation between log $\rho _{{{xy}}}^{\mathrm{A}}$ and log *ρ*_*xx*_ at low temperature region (Fig. 4c) can be fitted using the formula $\rho _{{{xy}}}^{\mathrm{A}} \propto \rho _{{{xx}}}^\alpha$, which gives the scaling exponent *α* = 1.87(4). It suggests that the intrinsic KL or extrinsic side-jump mechanism are mainly responsible for the AHE in Co_3_Sn_2_S_2_. On the other hand, the dominant mechanism of AHE can also be decided by fitting the relationship between $\rho _{{{xy}}}^{\mathrm{A}}$ and *ρ*_*xx*_ using the formula $\rho _{{{xy}}}^{\mathrm{A}}$ = $a(M)\rho _{{{xx}}} + b(M)\rho _{{{xx}}}^{\mathrm{2}}$. The first term corresponds to the skew scattering contribution, while the second term represents the intrinsic or side-jump contribution^40^. For the skew scattering contribution, the *a*(*M*) is usually proportional to *M* linearly^41^, and for the intrinsic contribution, the *b*(*M*) = $\rho _{{{xy}}}^{\mathrm{A}}{\mathrm{/}}\rho _{{{xx}}}^{\mathrm{2}}$ is directly related to the intrinsic AHC $\sigma _{{{xy,\mathrm{in}}}}^{\mathrm{A}} = - \rho _{{{xy}}}^{\mathrm{A}}{\mathrm{/}}\rho _{{{xx}}}^{\mathrm{2}}$. Previous study suggests that the $\sigma _{{{xy,\mathrm{in}}}}^{\mathrm{A}}$ is also proportional to *M* linearly^40^, thus the linear fit of $\rho _{{{xy}}}^{\mathrm{A}}{\mathrm{/}}\left( {M\rho _{{{xx}}}} \right)$ vs. *ρ*_*xx*_ can separate the intrinsic and extrinsic contributions. The relation between $\rho _{{{xy}}}^{\mathrm{A}}{\mathrm{/}}\left( {M\rho _{{{xx}}}} \right)$ and *ρ*_*xx*_ (Fig. 4d) shows the linear dependence when *ρ*_*xx*_ ≤ 250 μΩ cm. After subtracting the skew-scattering contribution, the obtained value of $| {\sigma _{{{xy,\mathrm{in}}}}^{\mathrm{A}}} |$ at 5 K is about 505 Ω^−1^ cm^−1^, which is comparable with the predicted value by using the formula of *σ*_*xy*_ = *Ke*^2^/4*π*^2^ (~525 Ω^−1^ cm^−1^) (Supplementary Fig. 6) but smaller than that calculated from the integral of Berry curvature along *k*_*z*_ in the BZ (~1310 Ω^−1^ cm^−1^) when setting the moment of Co as 0.33 μ_B_ (Fig. 2e). The discrepancy between experimental results and the theoretical results from the latter method is still not clear. But such kind of difference has also been observed in other systems, such as Fe [751 Ω^−1^ cm^−1^ (experiment) vs. 1032 Ω^−1^ cm^−1^ (theory)], and Ni [−2073 Ω^−1^ cm^−1^ (experiment) vs. −646 Ω^−1^ cm^−1^ (theory)]^14,42^. On the other hand, the $\sigma _{{{xy,\mathrm{in}}}}^{\mathrm{A}}$ exhibits the linear dependence of *M* when *T* < *T*_C_ (Fig. 4e), well consistent with the KL theory^10^. Importantly, this result is also well in agreement with the theoretical predictions that the smaller moment of Co leads to the nearly linear decrease of $\sigma _{{{xy}}}^{\mathrm{A}}$ (Fig. 2e and Supplementary Fig. 6). In contrast to the $| {\sigma _{{{xy,\mathrm{in}}}}^{\mathrm{A}}} |$, the $| {\sigma _{{{xy,\mathrm{sj}}}}^{\mathrm{A}}} |$ for the side-jump contribution can be estimated using an expression (*e*^2^/(*ha*)(*ε*_SO_/*E*_F_), where *ε*_SO_ is the spin–orbit interaction energy^41,43^. Using the lattice constant *a* ~ *V*^1/3^ = 9.96 Å and *ε*_SO_/*E*_F_ ~ 0.01 for the metallic ferromagnets, the derived $\sigma _{{{xy,\mathrm{sj}}}}^{\mathrm{A}}$ is only about 3.9 Ω^−1^ cm^−1^, thus the extrinsic side-jump contribution to $\sigma _{{{xy}}}^{\mathrm{A}}$ could be very small when compared to $| {\sigma _{{{xy,\mathrm{in}}}}^{\mathrm{A}}} |$. To further investigate the mechanism of AHE in Co_3_Sn_2_S_2_, the temperature dependence of $\sigma _{{{xy}}}^{\mathrm{A}}$ is shown in Fig. 4f. The $| {\sigma _{{{xy}}}^{\mathrm{A}}} |$ shows fairly large values at low temperature region and it is insensitive to the change of temperatures. Above 140 K, however, it decreases quickly with increasing temperature and reaches to about 150 Ω^−1^ cm^−1^ at 175 K. In contrast, the scale factor *S*_H_ ($= {\mathrm{\mu}} _0R_{\mathrm{s}}{\mathrm{/}}\rho _{{{xx}}}^{\mathrm{2}} = - \sigma _{{{xy}}}^{\mathrm{A}}{\mathrm{/}}M$) is almost constant when *T* < *T*_C_ (inset of Fig. 4f). Therefore it further confirms that the temperature dependence of $\sigma _{{{x}}y}^{\mathrm{A}}$ originates from the temperature dependence of *M*, i.e., the dramatic decrease of $| {\sigma _{{{xy}}}^{\mathrm{A}}} |$ above 140 K is mainly due to the sharp decrease of *M*(*T*) as the temperature approaches *T*_C_. Above results clearly indicate that the large intrinsic AHC is dominant in HMFM Co_3_Sn_2_S_2_, and it is closely related to the nontrivial topology of band structure with Weyl nodes near *E*_F_.

In summary, we have investigated the AHE and electronic structure of HMFM Co_3_Sn_2_S_2_ single crystal. The nearly quadratic relationship between $\rho _{{{xy}}}^{\mathrm{A}}$ and *ρ*_*xx*_ indicates that the mechanism of AHE in Co_3_Sn_2_S_2_ is dominated by the intrinsic contribution. The consistency between the experimental band structures and first-principles calculations, especially the spindle-shaped FSs along the $\overline {\mathrm{\Gamma }} - \overline M$ direction and the band dispersions forming the WPs, combined with the Weyl-cone structure presented in ARPES, indicate that the intrinsic AHE in Co_3_Sn_2_S_2_ originates from the existence of magnetic Weyl fermions near *E*_F_. Moreover, the steep decrease of $\sigma _{{{xy}}}^{\mathrm{A}}$ at high temperature can be mainly ascribed to the sharp decrease of magnetic moment, causing the change of topological properties in band structure. Current work will not only deepen our understanding on the exotic physical phenomena associated with nontrivial band topology, but also shed light on exploring novel electronic/spintronic devices based on AHE and/or half metallicity.

## Methods

### Single crystal growth and structural characterization

Co_3_Sn_2_S_2_ single crystals were grown by the Sn flux^44^. The starting materials Co lumps (purity 99.99%), Sn grains (purity 99.99%) and S flakes (purity 99.999%) were mixed together in a molar ratio of Co:Sn:S = 8:86:6 and placed in an alumina crucible. Then, the crucible was sealed in the quartz ampoule under partial argon atmosphere. The sealed quartz ampoule was initially heated at 673 K for 2 h in order to avoid bursting of ampoule due to the high vapor pressure of S. And then it was heated up to 1323 K and kept there for 6 h to ensure the homogeneity of melt. Finally the ampoule was cooled down slowly to 973 K with 5 K/h. At this temperature, the ampoule was taken out from the furnace rapidly and decanted with a centrifuge to extract Co_3_Sn_2_S_2_ single crystals from the Sn flux. X-ray diffraction (XRD) patterns were measured using a Bruker D8 X-ray machine with Cu*K*_*α*_ radiation (*λ* = 0.15418 nm) at room temperature.

### Magnetization and transport measurements

Magnetization and electrical transport measurements were performed in a Quantum design MPMS3 and PPMS, respectively. Both longitudinal and Hall electrical resistivity were measured using a standard four-probe method on rectangular shape single crystals with current flowing in the *ab* plane. In order to effectively avoid the longitudinal resistivity contribution due to voltage probe misalignment, the Hall resistivity was measured by sweeping the field from −5 T to 5 T at various temperatures, and the total Hall resistivity was determined by *ρ*_*xy*_(*μ*_0_*H*) = [*ρ*(+*μ*_0_*H*) − *ρ*(−*μ*_0_*H*)]/2, where *ρ*_*xy*_(±*μ*_0_*H*) is the transverse resistivity under a positive or negative magnetic field.

### ARPES measurements

ARPES measurements were performed at the Dreamline beamline of the Shanghai Synchrotron Radiation Facility (SSRF) with a Scienta D80 analyzer and at the 1-cubed ARPES end-station of BESSY using a Scienta R4000 analyzer. The energy and angular resolutions were set to 15 meV and 0.2°, respectively. All samples were cleaved in situ along the (001) plane and measured at *T* = 20 K in a working vacuum better than 5 × 10^−11^ Torr.

### Theoretical calculation

We have simulated the electronic structure of Co_3_Sn_2_S_2_ with first-principle calculations using Vienna ab initio Simulation Package (VASP)^45^, and the generalized gradient approximation (GGA) of Perdew–Burke–Ernzerhof (PBE) type exchange correlation potential^46^ was employed. The spin–orbit coupling was taken into account in all of the calculations. We have relaxed the crystal structure and the lattice constants *a* = *b* = 5.3892 Å, and *c* = 13.1519 Å are adopted in our calculation. The surface states and anomalous Hall conductivity were calculated based on the tight binding Hamiltonian constructed by using Wannier90 package^47^. The maximally localized Wannier functions for 3*d* orbitals on Co, 5*p* orbitals on Sn, and 3*p* orbitals on S have been used as the basis of the tight-binding Hamiltonian. We calculate the anomalous Hall conductivity (AHC) as the sum of Berry curvatures over all of the occupied bands^48^,

1 $$\sigma _{{{xy}}} = - \frac{{2\pi e^2}}{h}{\int}_{{\mathrm{BZ}}} \frac{{\mathrm{d}^3{\bf{k}}}}{{(2\pi )^3}}\mathop {\sum}\limits_n {\kern 1pt} f_n({\bf{k}}){\mathrm{\Omega }}_n^{\mathrm{z}}({\bf{k}})$$

where *f*_*n*_(**k**) is the Fermi–Dirac distribution function, and *n* is the index of the occupied bands. The Berry curvature can be arisen from the Kubo-formula derivation,

2 $${\mathrm{\Omega }}_n^{{z}}({\bf{k}}) = - 2{\mathrm{Im}}\mathop {\sum}\limits_{m \ne n} \frac{{\left\langle {{\mathrm{\Psi }}_{n{\bf{k}}}} \right|v_{{x}}\left| {{\mathrm{\Psi }}_{m{\bf{k}}}} \right\rangle \left\langle {{\mathrm{\Psi }}_{m{\bf{k}}}} \right|v_{{y}}\left| {{\mathrm{\Psi }}_{n{\bf{k}}}} \right\rangle }}{{\left( {E_m({\bf{k}}) - E_n({\bf{k}})} \right)^2}}$$

where *v*_*x*(*y*)_ is the velocity operator. The intrinsic AHC is calculated with 200 × 200 × 200 *k*-point grid based on the tight binding Hamiltonian.

## Electronic supplementary material

Supplemental Information

## Data Availability

Data measured or analyzed during this study are available from the corresponding authors on reasonable request.
