# Author Correction: Large intrinsic anomalous Hall effect in half-metallic ferromagnet Co_3_Sn_2_S_2_ with magnetic Weyl fermions

**DOI:** 10.1038/s41467-018-06643-x

**Published:** 2018-10-08

**Authors:** Qi Wang, Yuanfeng Xu, Rui Lou, Zhonghao Liu, Man Li, Yaobo Huang, Dawei Shen, Hongming Weng, Shancai Wang, Hechang Lei

**Affiliations:** 1 Department of Physics and Beijing Key Laboratory of Opto-electronic Functional Materials & Micro-nano Devices, Renmin University of China, 100872 Beijing, China; 2 Beijing National Laboratory for Condensed Matter Physics and Institute of Physics, Chinese Academy of Sciences, 100190 Beijing, China; 3 School of Physical Sciences, University of Chinese Academy of Sciences, 100190 Beijing, China; 4 State Key Laboratory of Functional Materials for Informatics and Center for Excellence in Superconducting Electronics, SIMIT, Chinese Academy of Sciences, 200050 Shanghai, China; 5 Shanghai Synchrotron Radiation Facility, Shanghai Institute of Applied Physics, Chinese Academy of Sciences, 201204 Shanghai, China; 6 Collaborative Innovation Center of Quantum Matter, Beijing, China

Correction to: *Nature Communications* 10.1038/s41467-018-06088-2; published online 11 September 2018

The original version of this article incorrectly omitted an affiliation of Hongming Weng: “Beijing National Laboratory for Condensed Matter Physics and Institute of Physics, Chinese Academy of Sciences, 100190, Beijing, China”

This has been corrected in both the PDF and HTML versions of the article.

