# Supplementary material for: Large intrinsic anomalous Hall effect in half-metallic ferromagnet Co3Sn2S2 with magnetic Weyl fermions
Source: Nat Commun. 2018 Sep 11;9:3681. doi: 10.1038/s41467-018-06088-2 (PMC6134149; doi:10.1038/s41467-018-06088-2)
Supplement: Supplementary file 1 — Supplemental Information [file 41467_2018_6088_MOESM1_ESM.pdf]

Supplementary Figures

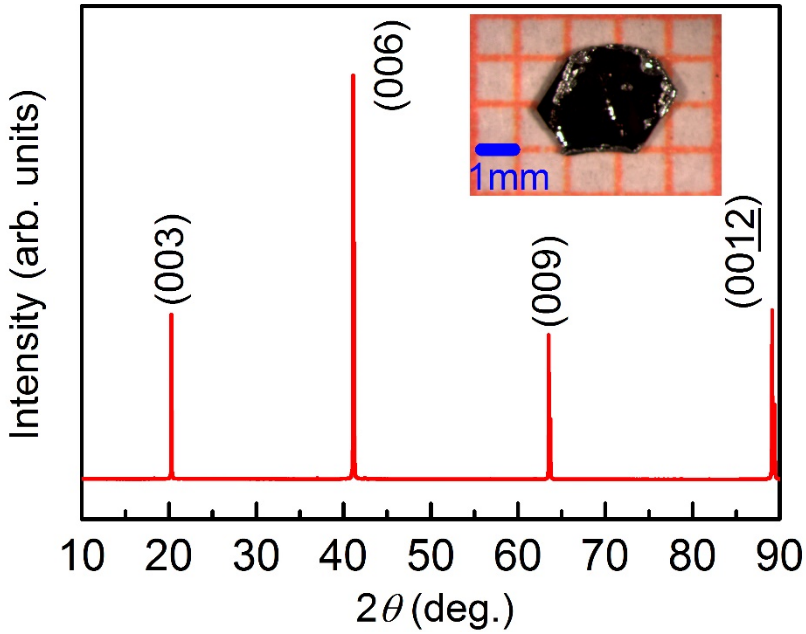

Supplementary Figure 1. XRD pattern of a $Co_3Sn_2S_2$ single crystal. All of peaks can be indexed by the indices of (00*l*) lattice planes. It reveals that the crystal surface is normal to the *c*-axis with the plate-shaped surface parallel to the *ab* plane. Inset: photo of typical $Co_3Sn_2S_2$ single crystals on a 1 mm grid paper.

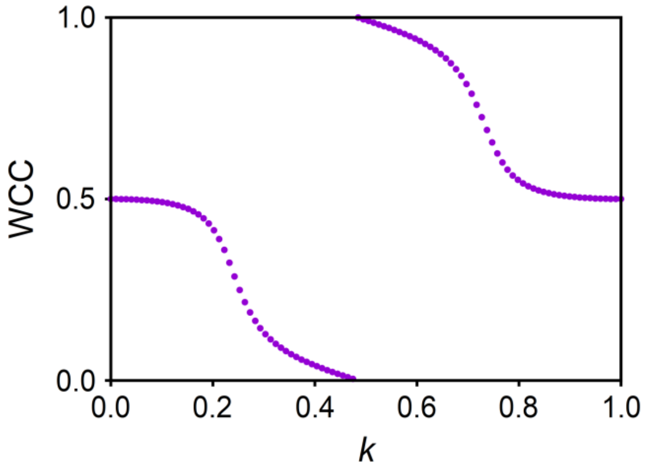

Supplementary Figure 2. The evolution of Wannier charge center (WCC) on a sphere that enclosing a WP in Brillouin Zone.

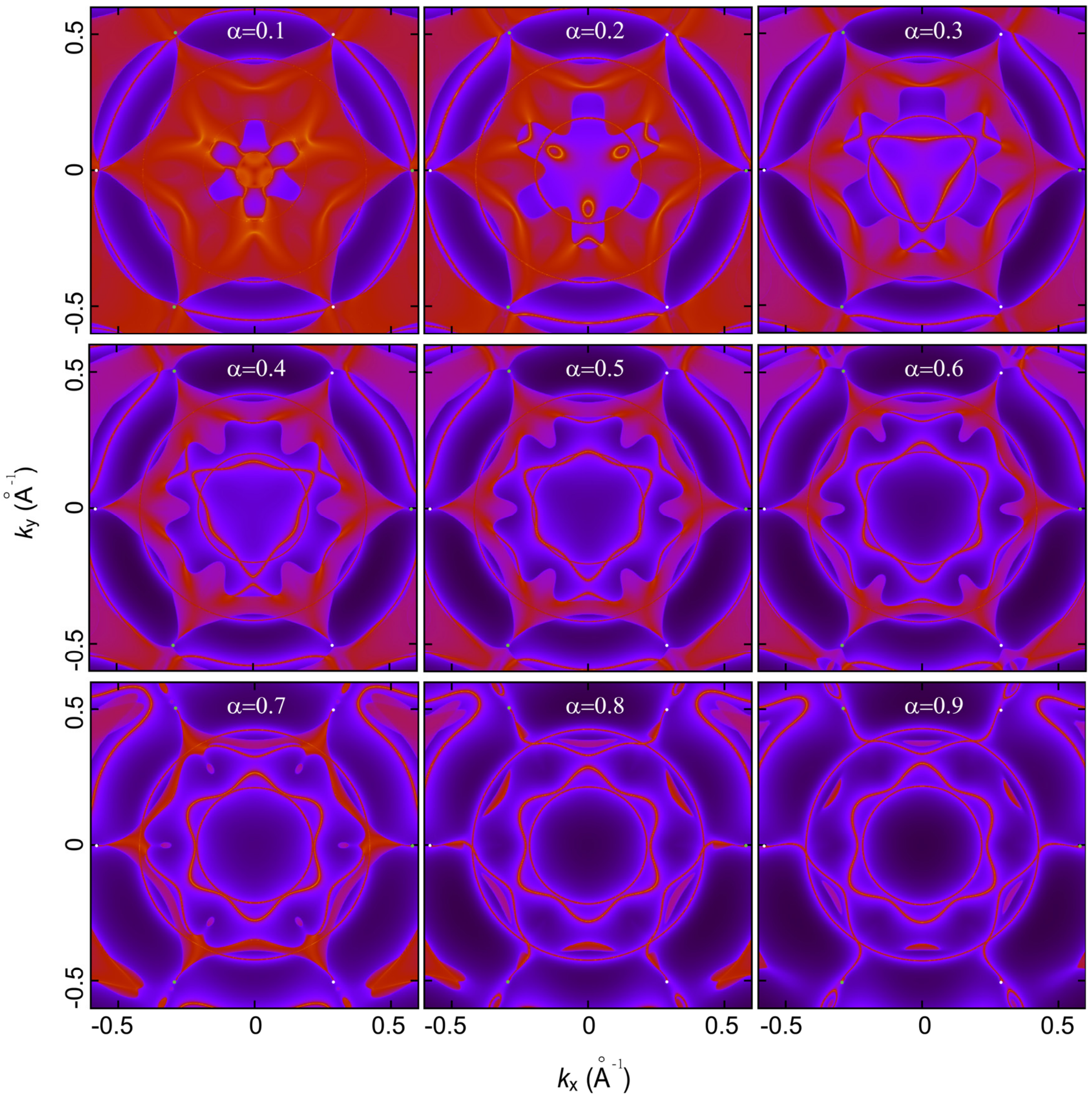

Supplementary Figure 3. Calculated (001) surface states of $Co_3Sn_2S_2$ cutting on the energy of WPs with the redefined Fermi energy $E_F(k) = \alpha[E_N(k) + E_{N+1}(k)]/2$.

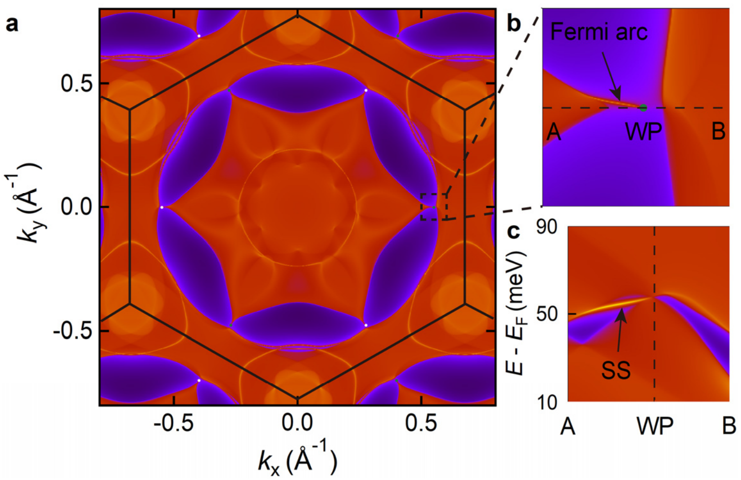

Supplementary Figure 4. Calculated (001) surface states of the $Co_3Sn_2S_2$ without structural relaxation. (**a**,**b**) Fermi surface calculation on the (001) surface cutting on the energy of WPs. The white and green dots represent the projected WPs with positive and negative chirality respectively. (**c**) The spectrum distribution near the WP along AB direction in **b**.

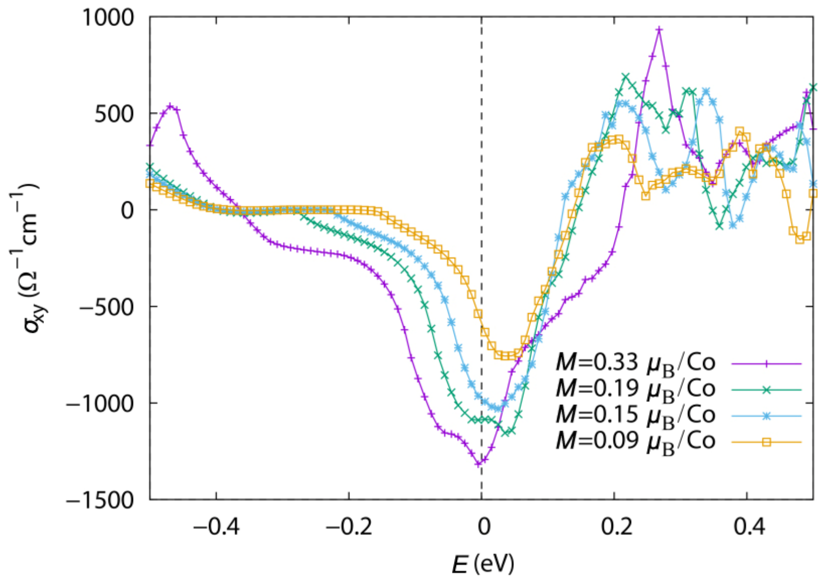

Supplementary Figure 5. Energy dependence of the intrinsic anomalous Hall conductivity with various magnetic moments of Co. The E_F is set to 0.

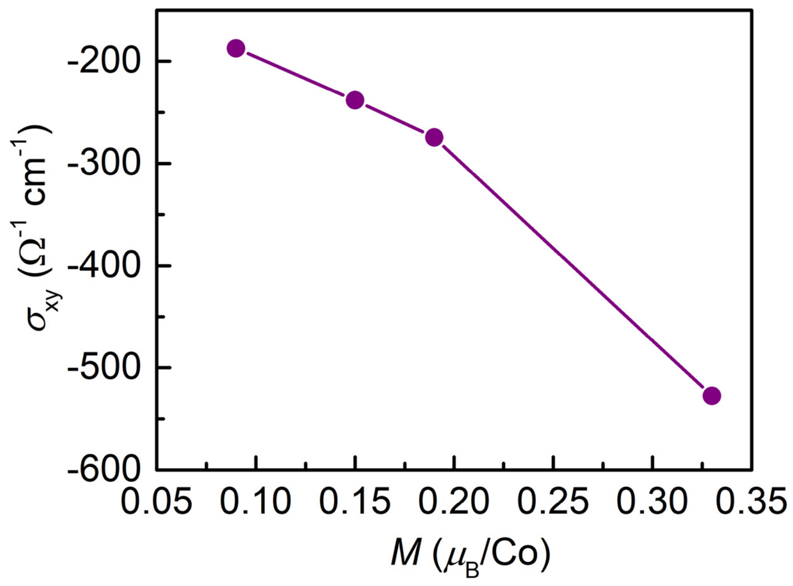

Supplementary Figure 6. The intrinsic AHC versus magnetization strength calculated by the formula of $\sigma_{xy} = Ke^2/4\pi^2$, where K is the distance along (001) direction between the Weyl points with opposite chirality.

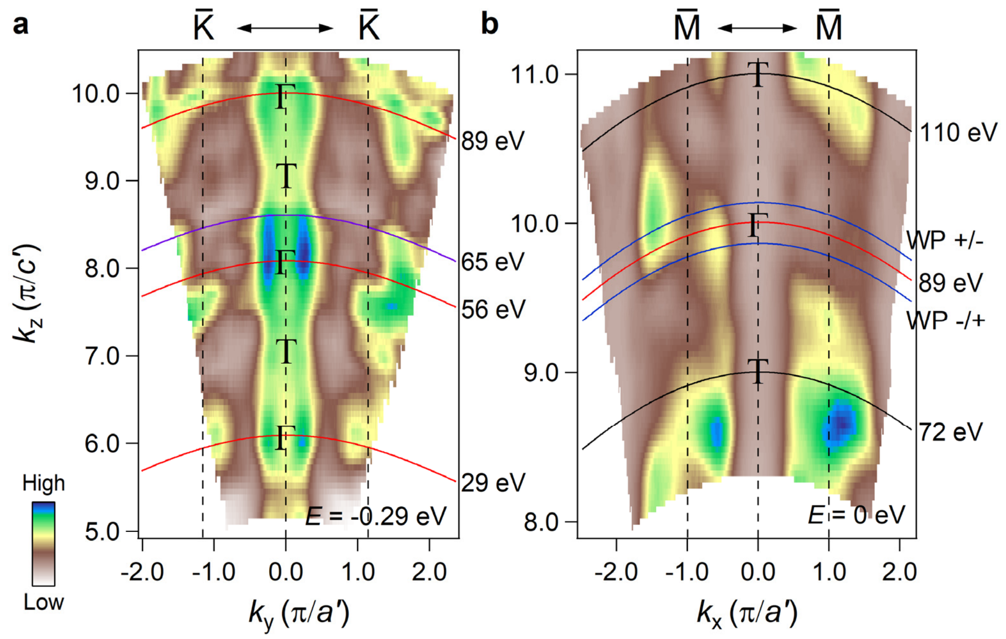

Supplementary Figure 7. Photon-energy-dependent study of $Co_3Sn_2S_2$. (**a**) ARPES intensity map at E = -0.29 eV in the k_y - k_z plane at k_x = 0 recorded with various photon energies, in which $a' = \sqrt{3}a/2$ (a = 5.38 Å), $c' = c/2$ (c = 13.19 Å). The inner potential (V_0) is estimated to be 10 eV. (**b**) Same as **a** but at E_F in the k_x - k_z plane at k_y = 0. Red and black curves indicate the momentum locations of k_z = 0 and π planes, respectively. Blue curves represent the $k_z = \pm\ 0.1455\ \pi$ planes, where the magnetic Weyl fermions with opposite chirality are located, respectively.

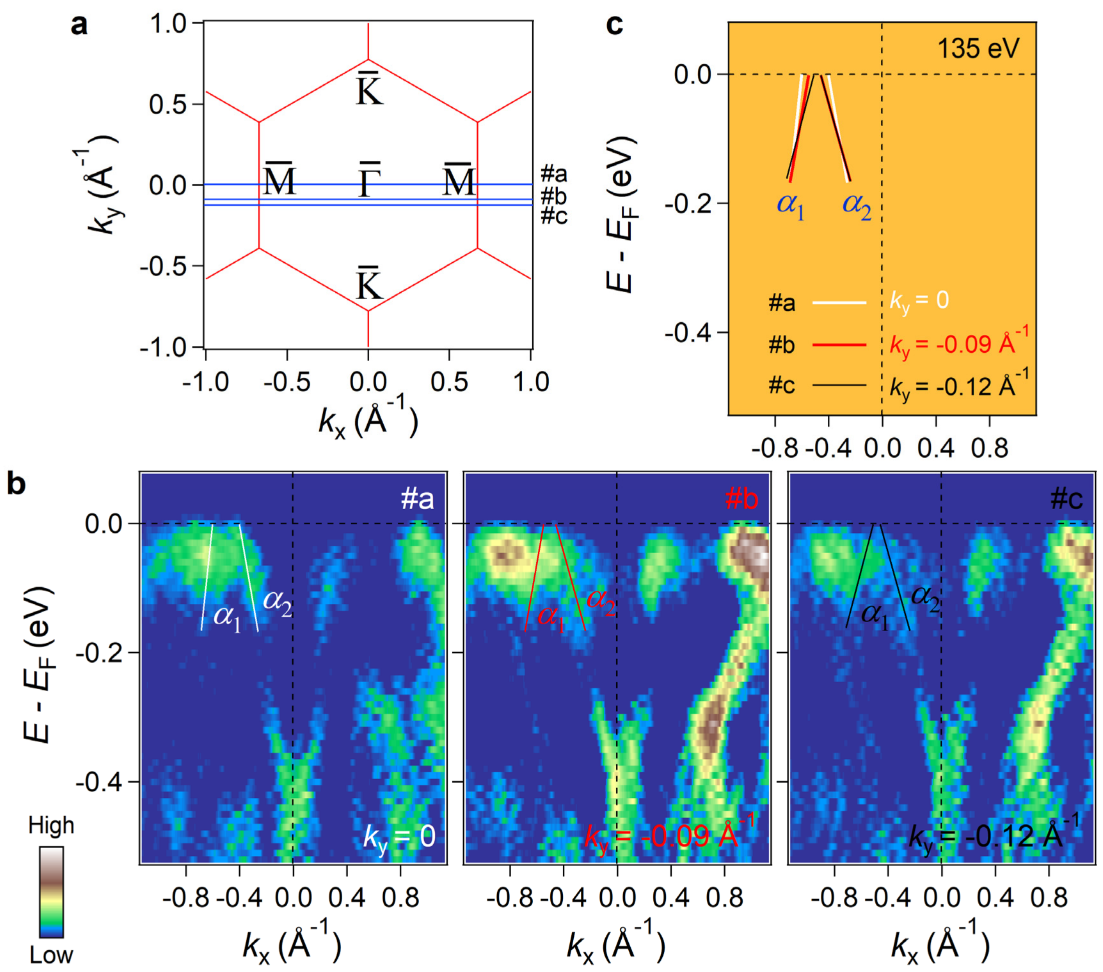

Supplementary Figure 8. Band structure evolution off the momentum position of Weyl point. (**a**) Schematic 2D BZs with marked cuts #a-#c indicating the momentum locations of the measured bands in **b**. (**b**) Second derivative intensity plots along cuts #a-#c in **a**, respectively, recorded at $h\nu = 135$ eV. The near-E_F α_1 and α_2 bands forming the Weyl nodes are traced out by solid lines. (**c**) Draft of the α_1 and α_2 band dispersions near E_F extracted from **b** for different k_y positions.

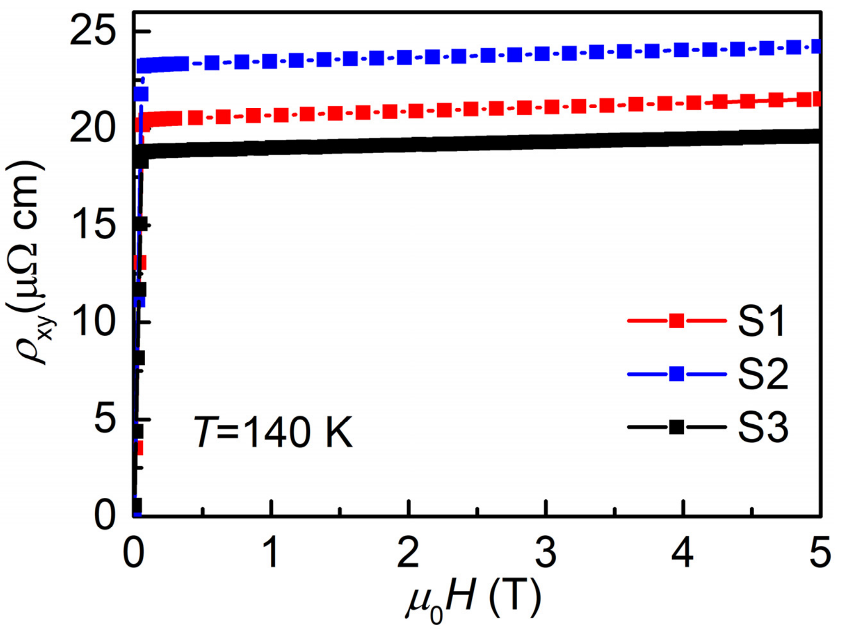

Supplementary Figure 9. Field dependence of Hall resistivity at 140 K for three crystals.

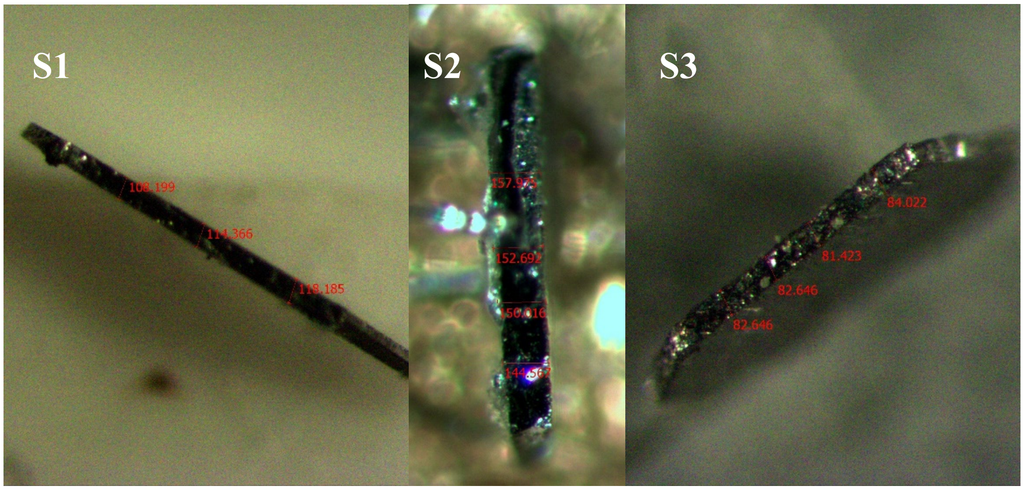

Supplementary Figure 10. Measurement uncertainty of sample thickness for three crystals. The unit of length is μm.

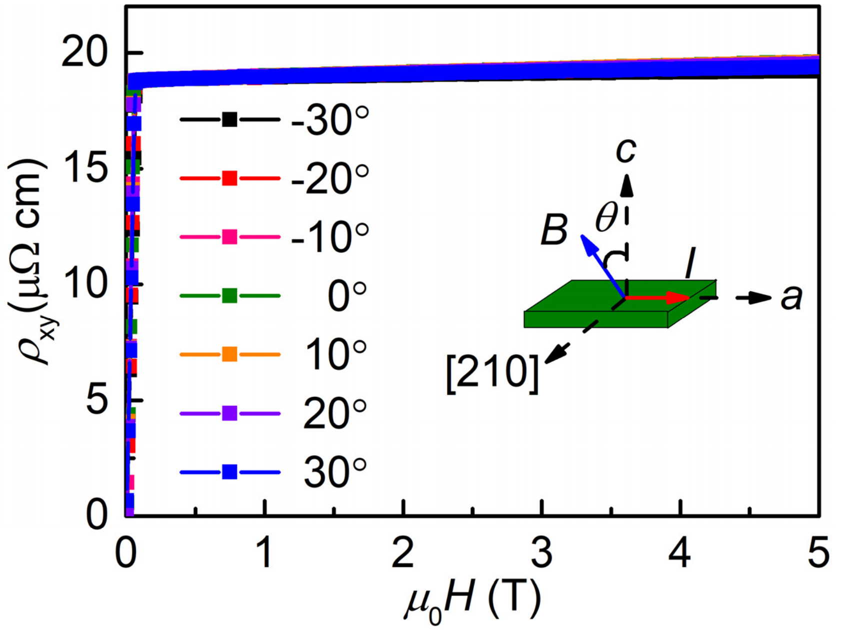

Supplementary Figure 11. Angular dependence of $\rho_{xy}(\theta)$. The θ is the angle between magnetic field and the *c* axis of $Co_3Sn_2S_2$ crystal. Magnetic field is always perpendicular to the current direction.

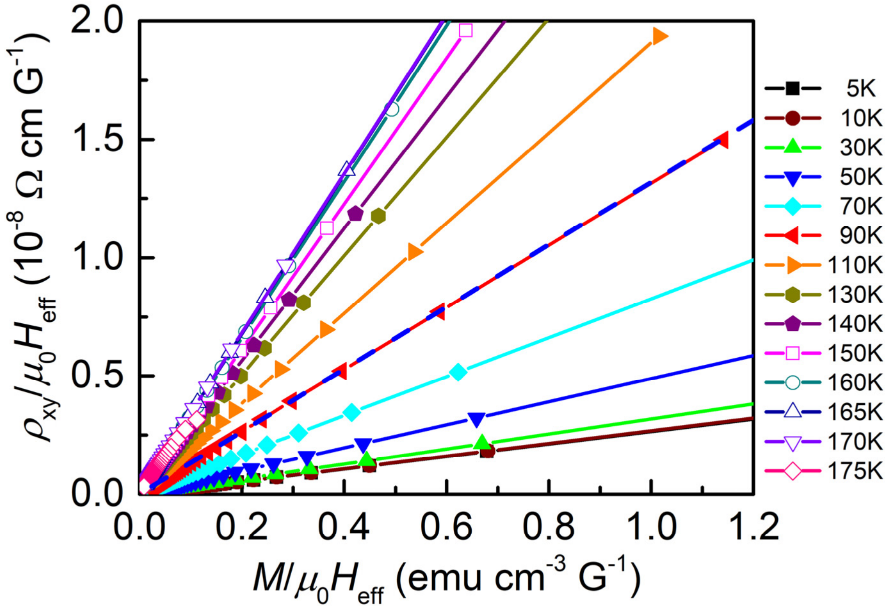

Supplementary Figure 12. ρ_{xy}/H_{eff} vs. M/H_{eff} at different temperatures. The blue dash line is the linear fit at 90 K.

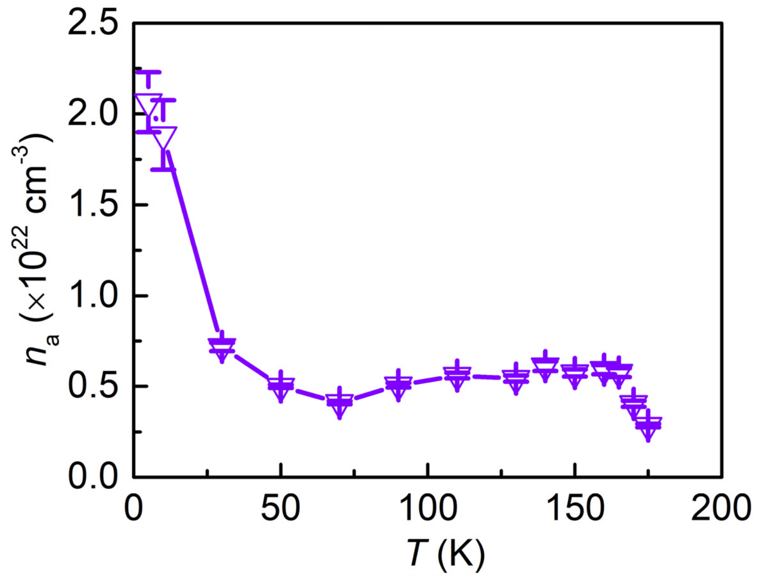

Supplementary Figure 13. Derived $n_a(T)$ from $R_0(T)$.

Supplementary Table

Supplementary Table 1. The positions and chirality of six pairs of Weyl points in the BZ. The positions (*k*1, *k*2, *k*3) are in unit of the reciprocal lattices.

No.	*k*1	*k*2	*k*3	Chirality
1	-0.059795	-0.059809	0.360922	+
2	-0.059795	0.360922	-0.059809	+
3	0.360922	-0.059795	-0.059809	+
4	0.059795	0.059809	-0.360922	-
5	0.059795	-0.360922	0.059809	-
6	-0.360922	0.059795	0.059809	-

Supplementary Note

Supplementary Note 1. For ferromagnets, $\rho_{xy} = \rho_{xy}^{O} + \rho_{xy}^{A} = R_0 B + 4\pi R_s M$, where ρ_{xy}^{O} is the normal Hall resistivity due to the Lorentz force, ρ_{xy}^{A} is the anomalous Hall resistivity. R_0 is the ordinary Hall coefficient, and R_s is the anomalous Hall coefficient. The induction field $B = \mu_0 H_{eff} + M$, where $\mu_0 H_{eff} = \mu_0 H - N_d M$ and N_d is the demagnetizing factor. The calculated value of N_d is 0.77. Substituting $B = \mu_0 H_{eff} + M$ and dividing both sides by $\mu_0 H_{eff}$, it can obtain $\rho_{xy} / \mu_0 H_{eff} = R_0 + (R_0 + R_s)(M/\mu_0 H_{eff})$. To extract R_0 and R_s, the relationships between $\rho_{xy} / \mu_0 H_{eff}$ and $M/\mu_0 H_{eff}$ at various temperatures from 5 K to 175 K are plotted (Supplementary Figure 12). The values of R_0 and R_s can be determined from the linear fittings of the curves. The slope and y-axial intercept is corresponding to the ($R_0 + R_s$) and R_0, respectively. Because the AHE vanishes above T_C, we are unable to accurately extract the R_0 and R_s by using this method at $T > T_C$.
